# *Klebsiella variicola* Is a Frequent Cause of Bloodstream Infection in the Stockholm Area, and Associated with Higher Mortality Compared to *K. pneumoniae*

**DOI:** 10.1371/journal.pone.0113539

**Published:** 2014-11-26

**Authors:** Makaoui Maatallah, Malin Vading, Muhammad Humaun Kabir, Amina Bakhrouf, Mats Kalin, Pontus Nauclér, Sylvain Brisse, Christian G. Giske

**Affiliations:** 1 Laboratoire d’Analyse, Traitement et Valorisation des Polluants de l’Environnement et des Produits, Faculté de Pharmacie, University of Monastir, Montasir, Tunisia; 2 Clinical Microbiology, MTC – Karolinska Institutet, Karolinska University, Hospital Solna, Stockholm, Sweden; 3 Department of Infectious Diseases, Karolinska University Hospital Solna, Stockholm, Sweden; 4 Institut Pasteur, Microbial Evolutionary Genomics, Paris, France; 5 CNRS, Paris, France; University of Mississippi Medical Center, United States of America

## Abstract

Clinical isolates of *Klebsiella pneumoniae* are divided into three phylogroups and differ in their virulence factor contents. The aim of this study was to determine an association between phylogroup, virulence factors and mortality following bloodstream infection (BSI) caused by *Klebsiella pneumoniae.* Isolates from all adult patients with BSI caused by *K. pneumoniae* admitted to Karolinska University Hospital, Solna between 2007 and 2009 (n = 139) were included in the study. Phylogenetic analysis was performed based on multilocus sequence typing (MLST) data. Testing for mucoid phenotype, multiplex PCR determining serotypes K1, K2, K5, K20, K54 and K57, and testing for virulence factors connected to more severe disease in previous studies, was also performed. Data was retrieved from medical records including age, sex, comorbidity, central and urinary catheters, time to adequate treatment, hospital-acquired infection, and mortality, to identify risk factors. The primary end-point was 30- day mortality. The three *K. pneumoniae* phylogroups were represented: KpI (n = 96), KpII (corresponding to *K. quasipneumoniae*, n = 9) and KpIII (corresponding to *K. variicola*, n = 34). Phylogroups were not significantly different in baseline characteristics. Overall, the 30-day mortality was 24/139 (17.3%). Isolates belonging to KpIII were associated with the highest 30-day mortality (10/34 cases, 29.4%), whereas KpI isolates were associated with mortality in 13/96 cases (13.5%). This difference was significant both in univariate statistical analysis (P = 0.037) and in multivariate analysis adjusting for age and comorbidity (OR 3.03 (95% CI: 1.10–8.36). Only three of the isolates causing mortality within 30 days belonged to any of the virulent serotypes (K54, n = 1), had a mucoid phenotype (n = 1) and/or contained virulence genes (*wcaG* n = 1 and *wcaG*/*allS* n = 1). In conclusion, the results indicate higher mortality among patients infected with isolates belonging to *K. variicola*. The increased mortality could not be related to any known virulence factors, including virulent capsular types or mucoid phenotype.

## Introduction

*Klebsiella pneumoniae* is a gram-negative pathogen causing a wide spectrum of both hospital- and community-acquired infections such as urinary tract infection, pneumonia, intraabdominal infection, bloodstream infection (BSI), meningitis and pyogenic liver abscess (PLA) [1]–[6]. The mortality in invasive infection is high, ranging between 17.5 and 23% [7]–[10].

Recently, *K. pneumoniae* has emerged as an increasingly resistant pathogen; it has shown an unprecedented ability to express several intrinsic and acquired mechanisms making the species frequently multidrug-resistant (MDR) to clinically important antimicrobial classes [11]. The clonal dissemination of resistant strains has been the focus of significant attention because of the lack of effective treatment options [12],[13]. Therefore, the dramatic increase in antibiotic-resistant outbreaks faced in several hospital and geographic locations has a high potential of causing mortality and morbidity [14]–[18].

*K. pneumoniae* displays a large spectrum of virulence factors associated with the infective potential of community-acquired-isolates [19]–[22]. The bacterial capsule is important for its virulence, with 78 capsular types identified [23],[24]. Among them K1, K2, K5, K20, K54, K57 were associated with invasive disease [6],[19],[21],[25]. Other potential pathogenic factors are the lipopolysaccharide [26],[27], iron scavenging systems, fimbrial and non-fimbrial adhesion factors [28],[29], hypermucoviscosity [30],[31], the plasmid-borne *rmpA* [32] (regulator of mucoid phenotype A), *wcaG*; encoding the capsular fucose synthesis, which may enhance the antiphagocytic activities [33], and *allS*; encoding the activator of allantoin regulon [34].

*Klebsiella* pneumoniae isolates display metabolic versatility, enabling bacteria of this genus to thrive in a variety of environmental niches [1],[20],[21],[35].

Phylogenetic analysis of clinical, carriage and environmental isolates classically identified as *K. pneumoniae* demonstrated the existence of three main phylogenetic lineages (phylogroups) called KpI, KpII and KpIII as initially demonstrated based on the sequence analysis of *gyrA* and *parC* genes [36],[37] and later by their association with specific families of chromosomal β-lactamase genes [38],[39]. From a taxonomical standpoint, *Klebsiella pneumoniae* comprises three subspecies [1],[36],[40],[41]: *K. pneumoniae* subsp. *pneumoniae*, *K. pneumoniae* subsp. *ozaenae* and *K. pneumoniae* subsp. *rhinoscleromatis*. The two latter subspecies are rarely encountered and are associated to specific diseases (rhinoscleroma and ozena, respectively). From a genetic viewpoint, these two subspecies represent homogeneous genotypic groups (MLST clonal complexes; CC) that are nested within the main phylogroup of *K. pneumoniae*, named KpI [36]. Subsequent taxonomic work has proposed the names *Klebsiella variicola*, for phylogroup KpIII [42] and *K. quasipneumoniae* for phylogroup KpII [43]. As a consequence, the name *K. pneumoniae* should now be used only for strains that belong to phylogroup KpI (*K. pneumoniae sensu stricto*).

It is difficult to distinguish *K. pneumoniae* from *K. quasipneumoniae* and *K. variicola* by biochemical tests [42],[44]. Until now, *K. variicola* and *K. quasipneumoniae* strains have therefore been generally misidentified as *K. pneumoniae* in clinical microbiology laboratories. Currently these three species can be more reliably differentiated by genotyping methods [36],[37],[45]. The introduction of mass spectrometry might offer a possibility of rectifying this problem in the future. As a result, the clinical importance of *K. variicola* and *K. quasipneumoniae* is currently underestimated, and there are few studies that have reported these species from clinical samples [37],[46]. *K. variicola* isolates were frequently isolated from various plants [42]. Previous investigations have shown that approximately 20% of human isolates thought to be *K. pneumoniae* are in fact *K. variicola*/KpIII or *K. quasipneumoniae*/KpII [37],[42].

A number of studies have shown association between pathogenicity of *K. pneumoniae* and virulence genes, serotypes, sequence types and mucoid phenotypes [21],[47]–[49]. The purpose of this study was to characterize clinical invasive isolates of *K. pneumoniae sensu lato* (i.e., in the classical sense and including *K. variicola, K. quasipneumoniae and K. pneumoniae sensu stricto*) from the Stockholm area and to analyze their population structure. A specific focus was to identify the main phylogroups. Further, our aims were to analyze their clonal diversity; to describe the association of virulence factors and serotypes with phylogroups; to study the association between phylogroups, bacterial traits and severity of disease, with primary endpoint being 30-day mortality.

## Materials and Methods

### Patients and bacterial isolates

All adult (≥18 years old) patients admitted to Karolinska University Hospital, Solna, Sweden, between 2007 and 2009, with growth of *K. pneumoniae* either in blood (n = 137) or cerebrospinal fluid (CSF) (n = 1) or both (n = 2) were included in this retrospective cohort study. Isolates and patients were detected by searches in the clinical microbiology laboratory information system. Species identification was done with API 20E system (bioMérieux, Marcy l’Etoile, France) or VITEK2 (bioMérieux). Antimicrobial susceptibility testing was performed with the disk diffusion method on Isosensitest agar (Oxoid, Basingstoke, UK) interpreted according to the guidelines of the Swedish Reference Group for Antibiotics (SRGA) [50]. Genotypic analysis of isolates producing extended-spectrum β-lactamases and/or carbapenemases was performed with Check-MDR (Checkpoints, Wageningen, The Netherlands). One isolate was excluded due to non-typability with MLST, hence the total amount of included isolates were 139. One blood or CSF isolate from each patient was used for the subsequent analyses.

### Capsule typing and virulence gene detection

Detection of serotypes and virulence genes was performed by PCR [51],[52]. We sought for six major serotypes that have been reported to be strongly associated with community-acquired invasive disease: K1, K2, K5, K20, K54, and K57. The virulence genes *allS, rmpA*, and *wcaG* were also investigated as described previously [21],[53]. Hypermucoviscous phenotype was determined using the string test, i.e. by pulling colonies with an inoculation loop, using a threshold of 5 mm [6].

### MLST

MLST was carried out as previously described [21],[54]. Updated details of protocol and primers for amplification and sequencing are given on the web site (http://bigsdb.web.pasteur.fr). Templates were sequenced on both strands with the published primers using the BigDye Terminator Ready Reaction Mix v3.1. Nucleotide sequences were determined by ABI Prism 3100 Genetic Analyzer (Applied Biosystems, Foster City, CA). New allelic variants were repeated and confirmed in triplicate. Alleles and profiles were determined by direct comparison with the *K. pneumoniae* MLST database. The alignment of sequences was performed with the Mega v5 [55]. Neighbor-joining trees were drawn for concatenate sequence of the seven MLST loci for the total population. To identify phylogenetic groups, three reference strains (group KpI: *K. pneumoniae* ATCC13883: ST3; group KpII: SB59: ST1118; and group KpIII: Kp342: ST146) were included in phylogenetic analyses [36]. The phylogenetic structure was also assessed by drawing separately a Neighbor-joining tree for each allele. Phylogenetic groupings were identified also by NeighborNet analysis based on the concatenated sequences using SplitsTree v4 [56].

DnaSP v5 [57] was used to calculate the nucleotide diversity of each gene for each phylogroup and for the whole population. To provide a graphical representation of the populations, a minimal spanning tree (MST) [58] was produced with BioNumerics v7.0 (Applied Maths, Sint Maartens-Latem, Belgium). Clonal complexes were defined as groups of isolates sharing six loci of their allelic profiles with at least one other member of the group, using the program eBURST v3 [59].

### Clinical parameters

The medical records were retrieved for the 139 cases, studying mortality, patient risk factors, hospital- versus community-acquired disease and antibiotic treatment. Charlson comorbidity index were constructed to assess comorbidity [60]. Infection was classified as polymicrobial if at least one more different species was recovered from specimens drawn within 24 h from the *K. pneumoniae* bloodstream infection. Skin contaminants (i.e., *Corynebacterium* spp., *Bacillus* spp., *Propionibacterium* spp., and coagulase-negative staphylococci [CoNS]) had to be present in at least two blood cultures, while for other species presence in one blood culture was sufficient. [61] Infections were defined as hospital-acquired if the sample was taken>48 h after admittance to hospital or if the patient had been admitted to hospital within the previous 30 days.

### Statistical analysis

Fisher’s test and Chi-squared test were used as appropriate for categorical variables. The Mann Whitney test was used to compare continuous variables. For all tests a two-sided p-value <0.05 was considered significant. Odds Ratio (OR) and 95% Confidence Intervals (CI) were calculated using logistic regression in the STATA 12.0 software. The multivariate analysis was restricted by the relatively few outcomes (mortality in analyzed groups, n = 23). Since our primary interest was to investigate the effect of phylogeny on mortality risk we decided to build a first model by including age and Charlson comorbidity index to adjust for host factors (these have previously been correlated to severe outcome in patients with severe bacterial disease). Both were adjusted for as continuous variables. In a second model we further included polymicrobial infection (present/absent) and gender. To avoid adjusting for factors that could be on the causal pathway between phylogroup and mortality, we did not adjust for disease manifestation (severity of disease and location). Time to adequate antibiotic therapy could be regarded as being on the causal pathway since it is likely influenced by disease manifestation. However, since it is often included in multivariate models of bacterial strains/clones and mortality we included time to adequate therapy as a continuous variable in a third model for comparison.

### Ethical considerations

Ethical approval for the study was obtained from the Karolinska Institutet Regional Ethics Committee of Stockholm (recordal 2009/1985-31/4). The committee approved that no written or verbal consent had to be given by the study subjects, as the study only pertained to extracting limited clinical data from patient charts. Patient records were anonymized and de-identified prior to analysis.

## Results

### Phylogenetic diversity and analysis of MLST data

**Figure 1:**
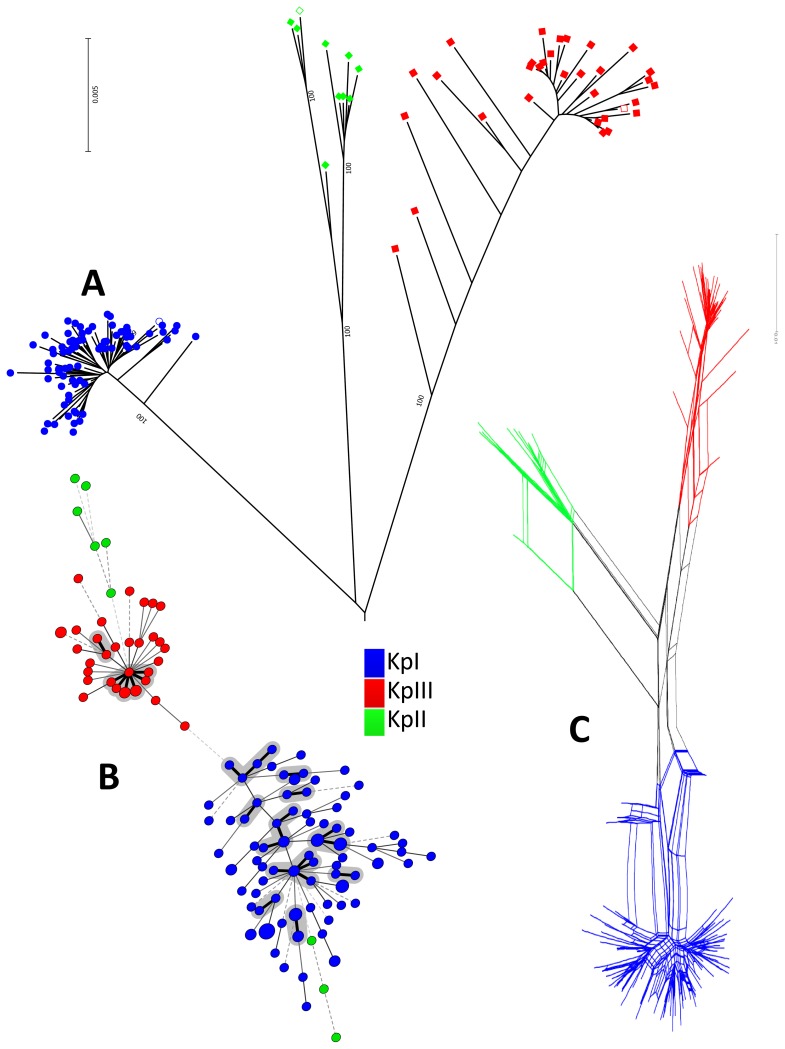
Phylogenetic representation of all isolates. **A**: Radial phylogenetic tree of 139 isolates based on the concatenated sequences of seven MLST loci performed using the Neighbor-joining method based on a Jukes-Cantor distance matrix. Main bootstrap values obtained are highlighted on at the main node of the phylogeny. The tree was rooted using the nucleotide sequences of the seven genes of *E. coli* and considered as an out-group. Each phylogroup was clearly separated from others. Colors of isolates symbols are specific of each phylogroups. Blue color corresponds to the KpI phylogroup, green corresponds to KpII, and red corresponds to KpIII. Empty symbols correspond to the references strains specific for each phylogroups. **B**: Minimal Spanning Tree (MST) analysis of *Klebsiella pneumoniae* strains based on MLST allelic profiles. Each circle corresponds to an ST. The area of each circle corresponds to the number of isolates. The relationships between strains are indicated by the connections between the isolates and the lengths of the branches linking them. Black lines connecting pairs of STs indicate that they differ in one allele (thick lines), two and three alleles (thin), or four to seven alleles (dashed). Colors of isolates symbols are specific to each phylogroup: blue - KpI, green - KpII and red - KpIII. Grey zones surround STs differing in one allele forming a clonal complex. **C**: NeighborNet graph based on seven concatenated housekeeping genes, illustrating the recombination within and among phylogroups. Colors surrounding each zone are specific of each phylogroup. Blue color corresponds to the KpI phylogroup, green corresponds to KpII, and red corresponds to KpIII.

The phylogenetic analysis of the concatenated sequences of the 7 MLST loci (Fig. 1A, Fig. S1) clearly revealed a well-defined structure, with three phylogroups that were each supported by maximal bootstrap values. The obtained phylogeny corroborates previous findings [36],[37],[42]. The three clades in the figures correspond to phylogroups KpI, KpII and KpIII (Fig. 1A, B, Fig. S1). KpI was the largest phylogroup consisting of 96 isolates, followed by KpIII (*Klebsiella variicola*) consisting of 34 isolates, and KpII (*K. quasipneumoniae*) consisting of 9 isolates. As previously noted [36], KpI and KpII were branched together while KpIII was branched in an external position (Fig. 1A). The mean genetic distance between clusters was 4.0% (KpI-KpII); 4.4% (KpI-KpIII) and 4.5% (KpII-KpIII). Furthermore the three phylogroups were confirmed by conducting a Neighbor-joining tree analysis with each of the individual alleles (data not shown). The NeighborNet graph (Fig. 1C) also demonstrated a clear demarcation among the three phylogroups and suggested a high level of recombination within each of the groups as well as among them.

The alignment of the seven housekeeping genes revealed the presence of minor events of insertion/deletion (INDEL) only in the *tonB* locus. The polymorphism parameters were assessed in the whole population, and in the three phylogroups (Table 1). Considering all isolates, the polymorphic sites ranged from 5.1% (*gapA*) to 19.6% (*tonB*), whereas the concatenate sequence revealed 10.8% polymorphic sites (Table 1). The average nucleotide diversity π was 2.1% for all the population. However, the internal level of π within the three phylogroups was much lower and the diversity within KpI (0.37%) and KpIII (0.63%) was lower than for KpII (1.3%). A ratio of Ka/Ks <1 was observed, as typical for housekeeping genes evolving predominantly under purifying selection.

**Table 1:** Nucleotide polymorphism within the seven housekeeping genes used for MLST**.**

Alleles	n	Sites	NetSites	S	Hap	Hd	π	θ	Ka/Ks
*gapA*	139	450	450	23	18	0.692	0.00819	0.009683	0.09948677
*infB*	139	318	318	28	19	0.756	0.015221	0.017127	0
*Mdh*	139	477	477	59	26	0.805	0.027967	0.023598	0.03242491
*Pgi*	139	432	432	54	28	0.693	0.02405	0.023955	0.01440774
*phoE*	139	420	420	41	41	0.939	0.014013	0.020316	0.00640582
*rpoB*	139	501	501	41	27	0.815	0.014476	0.015582	0.02302211
*tonB*	139	438	402	79	47	0.952	0.046675	0.040646	0.06934406
*Concatenate, Total 139 strains*	139	3036	3000	325	115	0.996	0.021386	0.021363	0.03443384
*Concatenate, KpI*	96	3024	3006	115	75	0.993	0.003715	0.007448	0.07781457
*Concatenate, KpII*	9	3018	3012	105	9	1	0.013206	0.012949	0.02047211
*Concatenate, KpIII (K. variicola)*	34	3018	3006	155	31	0.995	0.006379	0.012936	0.01251564

N = number of STs or samples subjected for analysis. Sites = length of sequences after alignments. Net Sites = length of sequences subjected for analysis (the gaps or missing data are rejected from analysis). S = polymorphic sites. Hap = haplotypes. Hd = haplotypic diversity. π = nucleotide diversity. θ = average number of nucleotide differences per site. Ka/Ks** = **ratio of the number of non-synonymous substitutions per non-synonymous site (Ka) to the number of synonymous substitutions per synonymous site (Ks).

The population analysis based on the MLST data showed an extensive haplotypic diversity, with 116 distinct STs. The eBURST and MST analyses (Fig. 1B) were both used to depict the clonal relatedness among sequence types (STs). The 139 isolates were subdivided into 12 clonal complexes comprising 48 isolates (Fig. 1B, Table 2). The remaining 91 isolates were divided into 79 singletons (STs that differed by two or more alleles from any other ST).

**Table 2:** eBURST analysis of the MLST data: illustration of clonal complexes based on STs and their allelic profiles.

Clonal Complexes	STs	Allelic profiles
CC347	347	16, 24, 21, 27, 47, 22, 67
	596	16, 24, 21, 27, 41, 22, 67
	595	16, 24, 21, 27, 55, 22, 67
	619	64, 24, 21, 27, 47, 22, 67
	197	16, 28, 21, 27, 47, 22, 67
	642	16, 24, 21, 24, 47, 22, 67
	597	16, 24, 21, 27, 47, 22, 105
CC253	253	2, 1, 1, 1, 9, 1, 13
	704	2, 1, 1, 1, 9, 1, 20
	163	2, 1, 1, 1, 9, 1, 12
	588	2, 1, 1, 1, 9, 1, 23
	618	2, 1, 1, 1, 116, 1, 13
CC25	25	2, 1, 1, 1, 10, 4, 13
	65	2, 1, 2, 1, 10, 4, 13
	280	2, 1, 2, 1, 10, 4, 46
	304	2, 1, 1, 1, 10, 4, 90
CC268	268	2, 1, 2, 1, 7, 1, 81
	703	16, 1, 2, 1, 7, 1, 7
	701	2, 1, 2, 2, 7, 1, 81
	36	2, 1, 2, 1, 7, 1, 7
CC17	17	2, 1, 1, 1, 4, 4, 4
	20	2, 3, 1, 1, 4, 4, 4
	336	2, 1, 1, 1, 72, 4, 4
CCI	504	2, 1, 1, 1, 3, 3, 18
	540	2, 1, 71, 1, 3, 3, 18
CCII	35	2, 1, 2, 1, 10, 1, 19
	693	2, 1, 2, 1, 10, 3, 19
CCIII	357	16, 24, 21, 27, 54, 22, 45
	599	16, 24, 21, 27, 111, 22, 45
CCIV	541	4, 1, 2, 1, 1, 1, 4
	584	4, 1, 2, 1, 1, 7, 4
CCV	15	1, 1, 1, 1, 1, 1, 1
	14	1, 6, 1, 1, 1, 1, 1
CCVI	13	2, 3, 1, 1, 10, 1, 19
	591	2, 3, 1, 1, 7, 1, 19
CCVII	593	2, 1, 2, 1, 9, 1, 16
	429	2, 1, 2, 1, 9, 1, 116

CCI to CCVII are an arbitrary designations of clonal complexes found in the present study, each complex formed only by two STs.

### Capsule types and virulence factors content of phylogroups

Among the 139 isolates (Fig. S2B, Table S1) only 18 strains (12.9%) were serotypable for the investigated capsular serotypes; K1 (1.4%; 2/139), K2 (5.0%; 7/139), K20 (1.4%; 2/139), K54 (2.2%; 3/139); K57 (2.9%; 4/139). K2 was the most frequently occurring serotype and K5 was not detected in any of the isolates. Of the 18 capsular types found in this study, 16 belonged to KpI and 2 to KpIII.

Regarding virulence genes (Fig. S2C, Table S1), *rmpA* was positive for 5 strains (3.6%; 5/139; ST25, 380, 592, 593 and 701), *wcaG* was positive for 12 strains (8.6%; 12/139; ST14, 29, 29, 581, 599, 604, 605, 616, 637, 695, 699 and 700) and *allS* was positive for 6 strains (4.3%; 6/139; ST104, 604, 605, 613, 620 and 702). A mucoid phenotype (Fig. S2D, Table S1) was detected in 8 isolates (5.8%; 8/139; ST20, 111, 268, 280, 380, 504, 542 and 592) out of which two were also positive for *rmpA*. With the exception of ST380, which was previously associated with *rmpA* [47], there were no other observed correlations between virulence genes and STs or clonal complexes. The 21 isolates with virulence genes were distributed as follows: KpI (n = 13), KpIII (n = 5) and KpII (n = 3).

### Association of clinical features with phylogroups

Among the 139 infectious episodes, 24 events were associated with a fatal outcome within 30 days translating to a total 30-day mortality of 17.3% (Table S1). Isolates belonging to phylogroup KpIII had the highest 30-day mortality (29.4%, 10/34 patients; Fig. S2A, Table S1). In phylogroup KpI and KpII the 30-day mortality was 13.5% (13/96) and 11.1% (1/9) (Fig. S2A, Table S1), respectively. In further comparisons of the association between phylogroup and mortality phylogroup KpII was excluded due to the small amount of patients. There was a significant difference in 30-day mortality between KpIII and KpI in crude analyses (OR 2.66 (95% CI: 1.04–6.82, p = 0.037) (Table 3). Increasing Charlson comorbidity index was strongly associated with mortality (p = 0.005). Metastatic cancer, which attributes 6 points to the Charlson comorbidity index, was also associated with mortality (p = 0.001). After adjustment for age and Charlson comorbidity index, KpIII remained associated with increased mortality (OR: 3.03 (95% CI: 1.10–8.36). Furthermore, after including age, Charlson comorbidity index, presence of polymicrobial infection and gender, as well as inclusion of time to adequate antibiotic therapy, KpIII was associated with increased mortality [OR: 3.11 (95% CI: 1.10–8.80) and 3.23 (95% CI: 1.12–9.27) respectively]. In a final analysis we excluded 7 patients that were treated at the intensive care unit at the time of onset of the BSI, which did not alter our results (KpIII adjusted OR: 3.53 (95% CI: 1.20–10.39).

**Table 3:** Comparison between patients with 30-day mortality and survivors.

	Dead within 30 days (n = 23) No. (%)	Survivors>30 days (n = 107) No. (%)	Dead vs. Survivors Significance P<0.05
**Clinical characteristics**			
Median age, years	73	68	0.35
Male sex	14 (60.9)	70 (65.4)	0.68
Charlson index (median)	5	2	**0.0003**
Charlson index 0–1	1 (4.4)	17 (15.9)	**0.005**
Charlson index 2–3	8 (34.8)	62 (57.9)	
Charlson index 4–5	3 (13.0)	12 (11.2)	
Charlson index>5	11 (47.8)	16 (15.0)	
Diabetes	2 (8.7)	20 (18.7)	0.25
Heart disease	8 (34.8)	28 (26.2)	0.40
Pulmonary disease	7 (30.4)	15 (14.0)	0.070
Kidney disease	4 (17.4)	18 (16.8)	1.00
Liver disease	6 (26.1)	16 (15.0)	0.22
CNS-disease	5 (21.7)	17 (15.9)	0.54
Intestinal disease	3 (13.0)	13 (12.1)	1.00
Malignancy, all	15 (62.5)	60 (56.1)	0.42
Hematological	3 (13.0)	21 (19.6)	0.57
Urogenital	5 (21.7)	18 (16.8)	0.56
Colorectal	1 (4.3)	10 (9.3)	0.69
Pulmonary	3 (13.0)	3 (2.8)	0.068
Bile/liver/pancreas	1 (4.3)	5 (4.7)	1.00
Miscellaneous	2 (8.7)	3 (2.8)	0.21
Metastasized	10 (43.5)	12 (11.2)	**0.001**
Previous organ transplant	1 (4.3)	2 (1.9)	0.45
Neutropenia	2 (8.7)	19 (17.8)	0.37
Hospital-acquired infection	11 (47.8)	54 (50.5)	0.82
Urinary catheter	9 (39.1)	41 (38.3)	0.94
Central catheter	7 (30.4)	33 (30.8)	0.97
Polymicrobial	8 (34.8)	24 (22.4)	0.21
* E coli*	5 (21.7)	9 (8.4)	0.074
Source of infection			
Urinary	7 (30.4)	47	0.23
Respiratory tract	3 (13.0)	5 (4.7)	0.15
Bile/liver	1 (4.3)	10	0.69
Gastrointestinal tract	3 (13.0)	6 (5.6)	0.20
Miscellaneous	3 (13.0)	10	0.59
Unknown	6 (26.1)	29 (27.1)	0.92
Time to adequate antimicrobial therapy			
0–1 h	2 (8.7)	12 (11.2)	0.98
1–2 h	6 (26.1)	21 (19.6)	
2–4 h	6 (26.1)	28 (26.2)	
4–24 h	6 (26.1)	33 (30.8)	
24–48	2 (8.7)	9 (8.4)	
>48 h	1 (4.4)	4 (3.7)	
**Bacterial characteristics**			
KpIII*	10 (43.5)	24 (22.4)	**0.037**
Mucoid phenotype	1 (4.3)	7 (6.5)	1.00
Serotypability	1 (4.3)	17 (15.9)	0.20
Virulence genes	1 (4.3)	17 (15.9)	0.20
Any virulence factor	2 (8.7)	29 (27.1)	0.060
ESBL	1 (4.3)	4 (3.7)	1.00

*Compared to KpI where the prevalence was 56.5% (13/23) in patients who died and. 77.6% (83/107) in patients who survived.

Among the 24 events associated with 30-day mortality only one isolate was serotypable (K54), and strikingly few (three isolates) featured virulence genes. Almost all isolates belonged to new sequence types, with the exception of ST15 (forming a clonal complex with ST14, which is prevalent among carbapenemase-producers) [53],[62],[63]; ST37 (previously described in bloodstream infection) [54]; ST337 (previously described in bloodstream infection in KPC-producing isolate, Colombia) [64],[65]; ST359 (previously described in bloodstream infection, colistin-resistant, South Korea) [66] and ST280 (previously described in bovine mastitis) (http://bigsdb.web.pasteur.fr). Table 4 summarizes patient characteristics. A majority of the patients (62.6%) were male, and the median age was 70 years. Comorbidity measured in Charlson comorbidity index ranged from 0 to 9. Most of the patients, 76/139 (54.7%) had an index of 2–3, 18 (24.5%), 15 (10.8%) and 30 (21.6%) had Charlson comorbidity index of 0–1, 4–5 and>5 respectively. The most common disease was having a malignancy with a prevalence of 81/139 (58.3%). Hematological malignancy was most common (n = 26) followed by urogenital malignancies and colorectal cancer (n = 25 and n = 11, respectively). 68/139 (48.9%) of the infectious episodes were considered hospital-acquired. Polymicrobial infections occurred in almost one fourth of the patients (34/139; 24.5%). The most frequent co-pathogen in the polymicrobial infections, observed in 15/139 (10.8%) patients, was *Escherichia coli* followed by *Staphylococcus aureus* n = 5 (3.6%) and *Enterococcus* spp, n = 5 (3.6%) (*E. faecalis* n = 2, *E. faecium* n = 2 and *E. casseliflavus* n = 1) respectively. There were no significant differences between the phylogenetic groups concerning patient characteristics (Table 4).

**Table 4:** Clinical characteristics of patients with invasive infection caused by *K. pneumoniae/variicola* **.**

	All (n = 139) No. (%)	KpI (n = 96) No. (%)	KpIII (n = 34) No. (%)	KpI vs. KpIII Significance P<0.05
Age, median, years	70	65.1	68	0.62
Male sex	87 (62.6)	58 (60.4)	26 (76.5)	0.093
Charlson index, median	2 (0–9)	2 (0–9)	3 (1–7)	0.37
Charlson index 0–1	18 (12.9)	17 (17.7)	1 (2.9)	0.07
Charlson index 2–3	76 (54.7)	47 (49.0)	23 (67.7)	
Charlson index 4–5	15 (10.8)	10 (10.4)	5 (14.7)	
Charlson index>5	30 (21.6)	22 (22.9)	5 (14.7)	
Diabetes	24 (17.3)	15 (15.6)	7 (20.6)	0.51
Heart disease	38 (27.3)	25 (26.0)	11 (32.4)	0.48
Pulmonary disease	25 (18.0)	14 (14.6)	8 (23.5)	0.23
Kidney disease	25 (18.0)	17 (17.7)	5 (14.7)	0.69
Liver disease	22 (15.8)	16 (16.7)	6 (17.6)	0.90
CNS-disease	24 (17.3)	18 (18.8)	5 (14.7)	0.60
Intestinal disease	17 (12.2)	13 (13.5)	6 (17.6)	0.56
Malignancy, all	81 (58.3)	51 (53.1)	21 61.8)	0.38
Hematologic	26 (18.7)	16 (16.7)	8 (23.5)	0.38
Urogenital	25 (18.0)	18 (18.8)	5 (14.7)	0.60
Colorectal	11 (7.9)	7 (7.3)	4 (11.8)	0.48
Pulmonary	7 (5.0)	4 (4.2)	3 (8.8)	0.38
Bile/liver/pancreas	6 (4.3)	6 (6.3)	0	0.34
Miscellaneous	6 (4.3)	3 (3.1)	2 (5.9)	0.61
Metastasized	26 (18.7)	18 (18.8)	5 (14.7)	0.60
Prev. organ transplant	4 (2.9)	3 (3.1)	0 (0)	0.57
Neutropenia	24 (17.3)	14 (14.6)	7 (20.6)	0.42
Hospital-acquired infection	68 (48.9)	50 (52.1)	14 (41.2)	0.27
Urinary catheter	54 (38.8)	40 (41.7)	10 (29.4)	0.21
Central catheter	43 (30.9)	28 (29.2)	12 (35.3)	0.51
Source of infection				
Urinary	59 (42.4)	42 (43.8)	12 (35.3)	0.39
Respiratory tract	8 (5.8)	6 (6.3)	2 (5.9)	1.00
Bile/liver	11 (7.9)	9 (9.4)	2 (5.9)	0.73
Gastrointestinal tract	9 (6.5)	4 (4.2)	5 (14.7)	0.052
Miscellaneous	14 (10.1)	11 (11.5)	2 (5.9)	0.51
Unknown	38 (27.3)	26 (27.1)	11 (32.4)	0.63
Polymicrobial (all)	34 (24.5)	21 (21.9)	11 (32.4)	0.22
* E. coli*	15 (10.8)	9 (9.4)	5 (14.7)	0.52
* S. aureus*	5 (3.6)	3 (3.1)	1 (2.9)	1.00
* Enterococcus spp*	5 (3.6)	2 (2.1)	2 (5.9)	0.28

### Antibiotic treatment and antimicrobial resistance

The most common antibiotic treatment, in 58.3% (81/139) of the patients, was cephalosporins (cefuroxime, cefotaxime or ceftazidime) (Table S1). Twenty-six patients (18.7%) received piperacillin-tazobactam as empiric treatment, while 13.7% (19/139) and 6.5% (9/139) started with a carbapenem (imipenem or meropenem) and ciprofloxacin, respectively. Aminoglycosides (gentamicin, amikacin) were added to the first dose of empiric treatment in 23/139 cases (16.5%) and in 4 of these cases this component was the only active against the *K. pneumoniae* isolate.

Within 4 hours, adequate antibiotic treatment was given to 77/139 patients (55.4%), 44/139 received adequate treatment within 2 hours (31.7%), and 15/139 (10.8%) within 1 hour (Table S1). After 24 hours 121/139 patients (87.1%) had received appropriate treatment, and after 48 hours most patients, 134/139 (96.4%) had received adequate antibiotic treatment.

Overall there was a low level of acquired antibiotic resistance (Table 5). The resistance levels against antimicrobials with relevant activities were as follows: trimethoprim/sulfametoxazole 10.8%, ciprofloxacin 8.6%, piperacillin/tazobactam 5.8%, gentamicin 3.6%, ceftazidime 4.3% and cefotaxime 3.6%. The resistance against trimethoprim/sulfametoxazole was significantly higher in KpI than compared to KpIII (P = 0.014). A total of 15.1% of the isolates were resistant to one or several of these antibiotic classes, 18.8% in KpI and 5.9% in KpIII (P = 0.078). These finding are similar to previous reports [37],[46] in which *K. variicola* isolates have been found less resistant than KpI and KpII for several classes of antimicrobial agents.

**Table 5:** No of isolates with non-susceptibility (intermediate and resistant) to antimicrobials.

Antimicrobial	All (139)	KpI (n = 96)	KpIII (n = 34)	KpI vs. KpIII Significance P<0.05
Ciprofloxacin	12 (8.6)	10 (10.4)	2 (5.9)	0.73
Trimethoprim/sulfametoxazole	15 (10.8)	15 (15.6)	0	**0.014**
Cefotaxime	5 (3.6)	5 (5.2)	0	0.33
Ceftazidime	6 (4.3)	5 (5.2)	0	0.33
Gentamicin	5 (3.6)	5 (5.2)	0	0.33
Piperacillin/tazobactam	8 (5.8)	8 (8.3)	0	0.11
Meropenem	1 (0.7)	1 (1.0)	0	1.00
Multidrug-resistance (I or R ≥3 classes of antibiotics)	5 (3.6)	5 (5.2)	0	0.33
ESBL* (including CPE)	5 (3.6)	5 (5.2)	0	0.33
CPE (ESBL_CARBA_)**	1 (0.7)	1 (1.0)	0	1.00
Total isolates with resistance to any group above	21 (15.1)	18 (18.8)	2 (5.9)	0.074

CPE = carbapenemase-producing *Enterobacteriaceae.*

*Two isolates CTX-M-1, one SHV-ESBL, one CTX-M-1+SHV-ESBL in genotypic analysis.

**VIM, CTX-M-1, CMY-2 in genotypic analysis.

Five of 139 isolates (3.6%) were considered multidrug-resistant (resistant to ≥three of the following antibiotic classes; trimethoprim/sulfametoxazole, fluoroquinolones, piperacillin/tazobactam, cephalosporins, aminoglycosides or carbapenems) [67]. These isolates all produced extended-spectrum β-lactamases (ESBL); two isolates CTX-M group 1 (ST15), one isolate CTX-M- group1 and an SHV-ESBL (ST340) and one isolate an SHV-ESBL (ST17). The fifth isolate (ST383) co-produced a CTX-M group 1 enzyme, CMY II, and the carbapenemase VIM. The patient had previously been hospitalized in Greece [68]. All ESBL-producing isolates belonged to KpI. Among the 4 patients with carbapenem susceptible ESBL-producing isolates, two received adequate empiric antibiotic treatment within 4 hours (carbapenems), in the other two cases there was a delay between 24 and 72 hours until adequate treatment was received (no death occurred). The patient with BSI caused by a VIM-producing strain received adequate antibiotic treatment, colistin, rifampicin and meropenem, after 4 days and had a fatal outcome. However this patient also had significant comorbidities requiring ICU-care, respirator and dialysis before the onset of the bloodstream infection.

## Discussion

To our knowledge this study is the first merging phylogroup classification, clinical perspective, virulence factors and sequence types on an inpatient consecutive material of bloodstream infections caused by *Klebsiella pneumoniae* (*sensu lato*) during a long time period and in a well-defined geographical area. The population structure of *K. pneumoniae* has been suggested to consist of three phylogroups [36] but this has not been thoroughly explored in specific geographical locations and with respect to clinical outcome and patient characteristics. According to our findings, the Swedish *K. pneumoniae* population comprises the three main phylogroups (Fig. 1A, B, C). KpI was the most frequent group consisting 96 isolates (69%), followed by KpIII consisting of 34 isolates (24%) and KpII consisting of 9 isolates (6%). Previously [37], 420 isolates from 26 European hospitals were classified as follows: 345 isolates (82.1%) identified as KpI, 29 (6.9%) as KpII and 46 (11%) as KpIII. Hence, the population in this study was comparable, in terms of phylogroup relative frequencies, to a Europe-wide sampling [37]. Phylogroups KpII and KpIII were given species status as *K. quasipneumoniae* and *K. variicola* [42],[43]. These observations confirm that *K. pneumoniae*, as defined biochemically and identified in clinical microbiology laboratories, must be regarded as a complex of three species. These three species appear to have wide geographic distribution and to be represented, among isolates initially identified as *K. pneumoniae*, in similar relative order of frequency in distinct geographical locations [46],[52].

Phylogroup KpI has been shown to be overrepresented among clinical *K. pneumoniae* isolates in previous studies [36],[46]. Contrary to previous studies, phylogroup KpIII (*K. variicola*) isolates were relatively more common in this study. Recently Seki et al. [69] described a case of fatal bloodstream infection caused by *K. variicola*. Initially, based on the classic and automated identification methods, the isolate was incorrectly identified as *K. pneumoniae*, but reclassified as *K. variicola* according to data derived from next-generation sequencing. As it is difficult to distinguish *K. variicola* from *K. pneumoniae* by classical methods used in clinical laboratories for species determination, *K. variicola* has been underreported in the literature. *K. variicola* was initially shown to be adonitol negative unlike *K. pneumoniae* [36],[42], but this test alone cannot reliably identify *K. variicola* [43],[46]. Utilization of 5-keto-D-gluconate was recently found to be specific of *K. variicola* and may represent a useful feature for identification [43]. Also, the usefulness of mass spectrometry for species identification of *K. variicola* remains to be determined.

Here we addressed for the first time to our knowledge, the possible presence of a link between phylogroup and bacterial traits (virulence factor, serotypes) or severity of disease. The overall 30-day mortality was 17.3%, similar to some of the previous studies on BSI caused by *K. pneumoniae* [7]–[10]. Analyzing phylogroups in relation to mortality, KpIII had the highest 30-day mortality (10/34, 29.4%). To the best to our knowledge, this is the first report indicating a relatively high mortality associated with *K. variicola.* The difference in mortality between KpI and KpIII was significant (P = 0.037) both in univariate analysis and in multivariate analysis adjusting for age, comorbidity, gender, time to adequate antimicrobial therapy, and polymicrobial infections. The difference in mortality between the clades cannot be explained by differences in clinical characteristics of patients since they were similar (Table 4). Measuring time to adequate antimicrobial treatment and other supportive therapy is complicated in this type of study as severity of disease and presentation of disease often influences choice and timing of empirical treatment. However as resistance rates were low in our study more than half of the patients (55.4%) received adequate antibiotic treatment within 4 hours. After 24 hours most patients (87.1%) had received adequate antibiotic treatment, and there were no differences among the survivors and the deaths in time to adequate antibiotics.

However there are limitations to consider. The major concern is the small number of events (30-d mortality of 23 patients in the two analyzed phylogroups), causing limitations in the multivariate model. Despite this concern, it is interesting that our data suggest that the underestimated pathogen *K. variicola* is associated with poor prognosis in BSI. The 30-d mortality is often used as an endpoint in studies measuring mortality. However, when studying BSI and mortality, there is always a risk that the cause of death is not attributable to the BSI, although this risk would be regarded equally large in all phylogroups. It could be debated whether other variables could be adjusted for, but the risk of this strategy is that such variables could be on the causal pathway between phylogroup and mortality.

Numerous hospital-based studies have notified that several comorbidities such as malignancy, diabetes mellitus, cirrhosis, biliary tract disorders and alcoholism may impair patient defenses and hence contribute to the development of *K. pneumoniae* infection [1],[7],[9],[70]–[73]. Previous studies on BSI caused by *K. pneumoniae* have shown that the disease often affects older patients with high comorbidity. Our study corroborates these earlier findings. Most of the patients in our study were male with a median age of 70, 32.4% had Charlson comorbidity index 4 or more, and almost half of the episodes were hospital acquired. Increasing Charlson comorbidity index was strongly associated with mortality (P = 0.005). Advanced age was associated with an increased, but not significant, risk of death. There was a similar amount of hospital-acquired infections among the deaths and survivors.

In accordance to what was reported previously [37],[46], our study demonstrates that isolates belonging to the phylogroup KpI were found to be more resistant than *K. variicola* isolates. From the present study, we retrieved only five MDR isolates that were ESBL-producers and belonged to KpI. Recently, Valverde et al. [74] determined the phylogenetic structure of a collection of ESBL-producing *K. pneumoniae* isolates. Expectedly, most of the ESBL-producing isolates belonged to KpI. A minority of isolates was classified in the KpIII group, and almost all of them were CTX-M-10 producers. Lastly, the prevalence of class 1 integrons was screened among these three phylogroups; the class 1 integrons were dominantly associated with KpI but very few were observed in isolates of KpIII [75]. As suggested previously, the high prevalence of KpI isolates among clinical isolates could be a reflection of the higher antimicrobial resistance rates within this group.

This collection of *K. pneumoniae* isolates contained few virulence factors compared with reports from previous studies, based on community-acquired BSI and severe disease. Mucoid phenotype (n = 8), virulence genes *rmpA*, *wcaG and allS* (n = 21 isolates; two of them had both *wcaG* and *allS*), and capsular serotypes K1, K2, K5, K20, K54 and K57 (n = 18) were all relatively rare in the collection. Much effort has been made in the last decades to estimate the link between the capsular types, virulence potential, and the nature of diseases, but mostly with community-acquired isolates, which differ in ST and virulence factors from nosocomial isolates [6],[21],[22],[31],[76]. Within the phyologroups in our dataset, there was no correlation between the serotypes or virulence genes and CCs or STs.

Previous reports have expressed concern about numerous bacterial virulence genes which were significantly associated with invasive strains. Herein, we show that *rmpA* gene was associated only to K2, K20 and K57, and not to K1, K5 and K54 as found in previous reports [51],[77]. In the same reports *wcaG*, encoding capsular fucose production, was associated with capsular types K1 and K54 [51],[77], which was further corroborated by our present results. Although the presence of the *wcaG* gene has not been well studied, there is data to suggest that strains harbouring this gene are more often observed in severe disease [51]. Interestingly, two fatal strains harboring the *wcaG* gene were recorded in our study (Table S1); ST616 (K54, *wcaG*, KpIII) and ST604 (*wcaG/allS*, KpII). Conversely, the six isolates harboring the *allS* gene did not belong to any of the investigated capsular types. Noteworthy, this gene was not universally associated to K1 serotype, but considered as a specific marker for the hypervirulent clone (CC23 K1) isolated from patients with pyogenic liver abscesses detected in several countries [21],[34],[47],[51],[76],[78]–[80]. In a recent report from France, five cases of fatal BSI were attributed to *K. pneumoniae* serotype K2, possessing the *rmpA* gene [47]. The isolates belonged to two clones; ST86 (two isolates) and ST380 (3 isolates). The same genotype (ST380, K2, *rmpA*) was encountered in our study but was not associated with a fatal outcome. In the 30-day mortality group (24/139 patients) only three isolates featured one or more virulence factors. In this study, where both community- and hospital-acquired BSIs were included, no indication for an existing correlation between above mentioned virulence factors and increased risk of mortality in BSI caused by *K. pneumoniae* could be found.

## Conclusions

We have analyzed the population structure of invasive clinical isolates of *K. pneumonia sensu lato* in Sweden, and found *K. pneumoniae* (KpI), but also *K. quasipneumoniae* (KpII) and *K. variicola* (KpIII). The 30-d mortality was more commonly associated with infecting strains belonging to *K. variicola*, but was not associated with known virulence factors, including some of the most virulent capsular types. More studies with enhanced species identification would be valuable to expand the data on the clinical importance of *K. variicola* in relation to *K. pneumoniae sensu stricto*. In general, a high level of comorbidity, equally high among the patients with BSI caused by *K. pneumoniae* as among the patients with BSI caused by *K. variicola*, was observed.

## Supporting Information

Figure S1 **Circular Neighbor-joining tree of 139 isolates based on the concatenated sequences of seven MLST loci based on a Jukes-Cantor distance matrix.** Main bootstrap values obtained are highlighted on at the main node of the phylogeny. The tree was rooted using the nucleotide sequences of the seven genes of *E. coli* and considered as an out-group. The tree displays all the STs into their phylogroups. Colors of isolates symbols are specific of each phylogroups. Blue color corresponds to the KpI phylogroup, green corresponds to KpII, and red corresponds to KpIII. Empty symbols correspond to the references strains specific for each phylogroups.
(TIF)

Figure S2 **Minimal Spanning Trees (MSTs) analysis of** ***Klebsiella pneumoniae*** **strains based on MLST allelic profiles.** Each circle corresponds to an ST. The area of each circle corresponds to the number of isolates. The relationships between strains are indicated by the connections between the isolates and the lengths of the branches linking them. Black lines connecting pairs of STs indicate that they differ in one allele (thick lines), two and three alleles (thin), or four to seven alleles (dashed). Four MST graphs were generated separately based on the following associations. A: MST vs mortality, B: MST vs serotypes, C: MST vs virulence genes and D: MST vs mucoid phenotype.
(TIF)

Table S1 **Database displaying the genotypic and phenotypic features of the studied strains.** Strains ID, MLST STs, Allelic profiles, phylogroups, patient sex, patient age, death within 30 days: 1 = yes, malignancy, Charlson comorbidity index, nosocomial (>48 h after admittance or < = 30 days after discharge), time to adequate antimicrobial therapy, mucoid phenotype, serotype and virulence factors.
(XLSX)
