# Supplementary figures and images for: Klebsiella variicola Is a Frequent Cause of Bloodstream Infection in the Stockholm Area, and Associated with Higher Mortality Compared to K. pneumoniae
Source: PLoS One. 2014 Nov 26;9(11):e113539. doi: 10.1371/journal.pone.0113539 (PMC4245126; doi:10.1371/journal.pone.0113539)

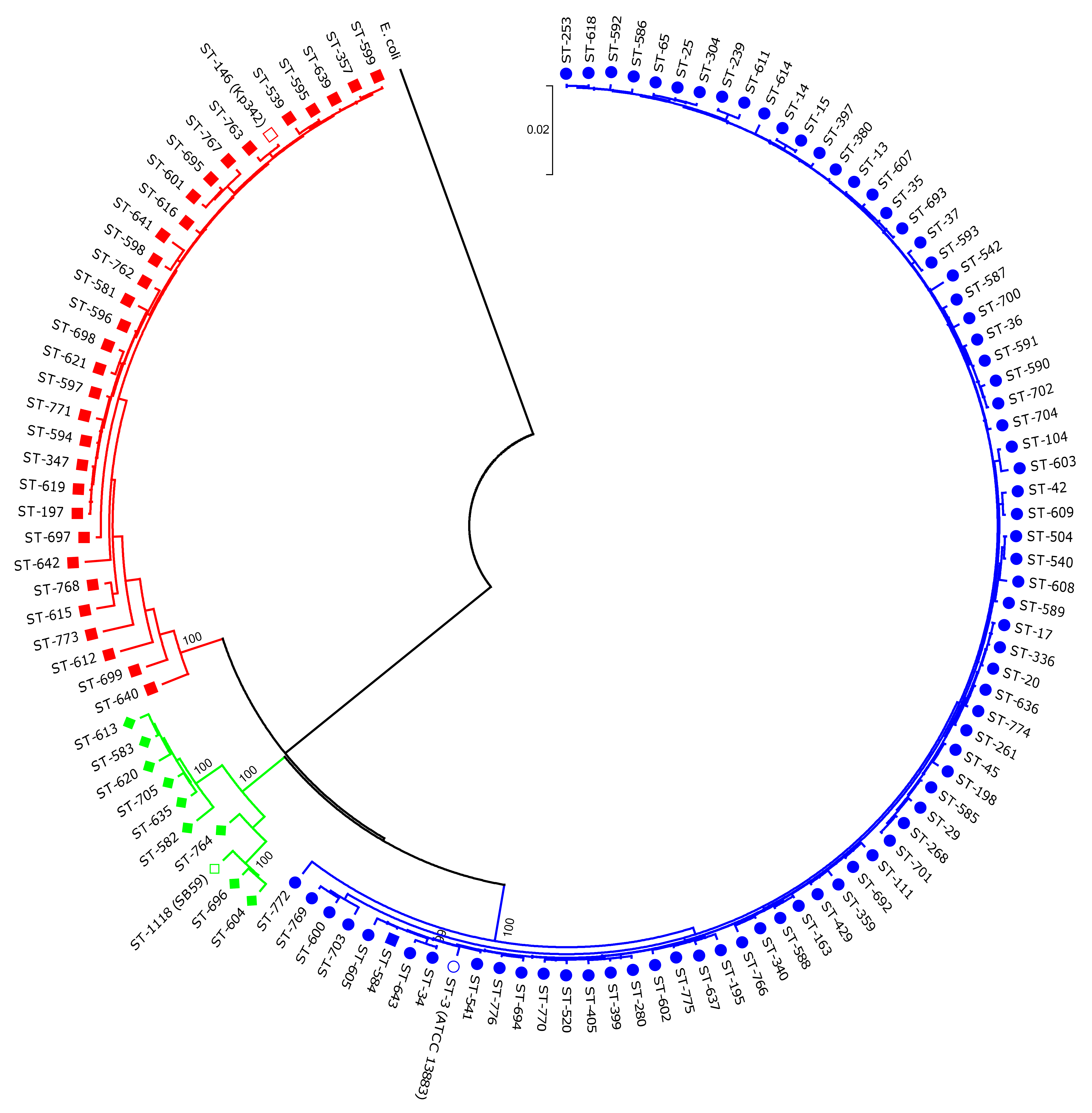

Supplement: Figure S1 — Circular Neighbor-joining tree of 139 isolates based on the concatenated sequences of seven MLST loci based on a Jukes-Cantor distance matrix. Main bootstrap values obtained are highlighted on at the main node of the phylogeny. The tree was rooted using the nucleotide sequences of the seven genes of E. coli and considered as an out-group. The tree displays all the STs into their phylogroups. Colors of isolates symbols are specific of each phylogroups. Blue color corresponds to the KpI phylogroup, green corresponds to KpII, and red corresponds to KpIII. Empty symbols correspond to the references strains specific for each phylogroups. (TIF) [file pone.0113539.s001.tif]

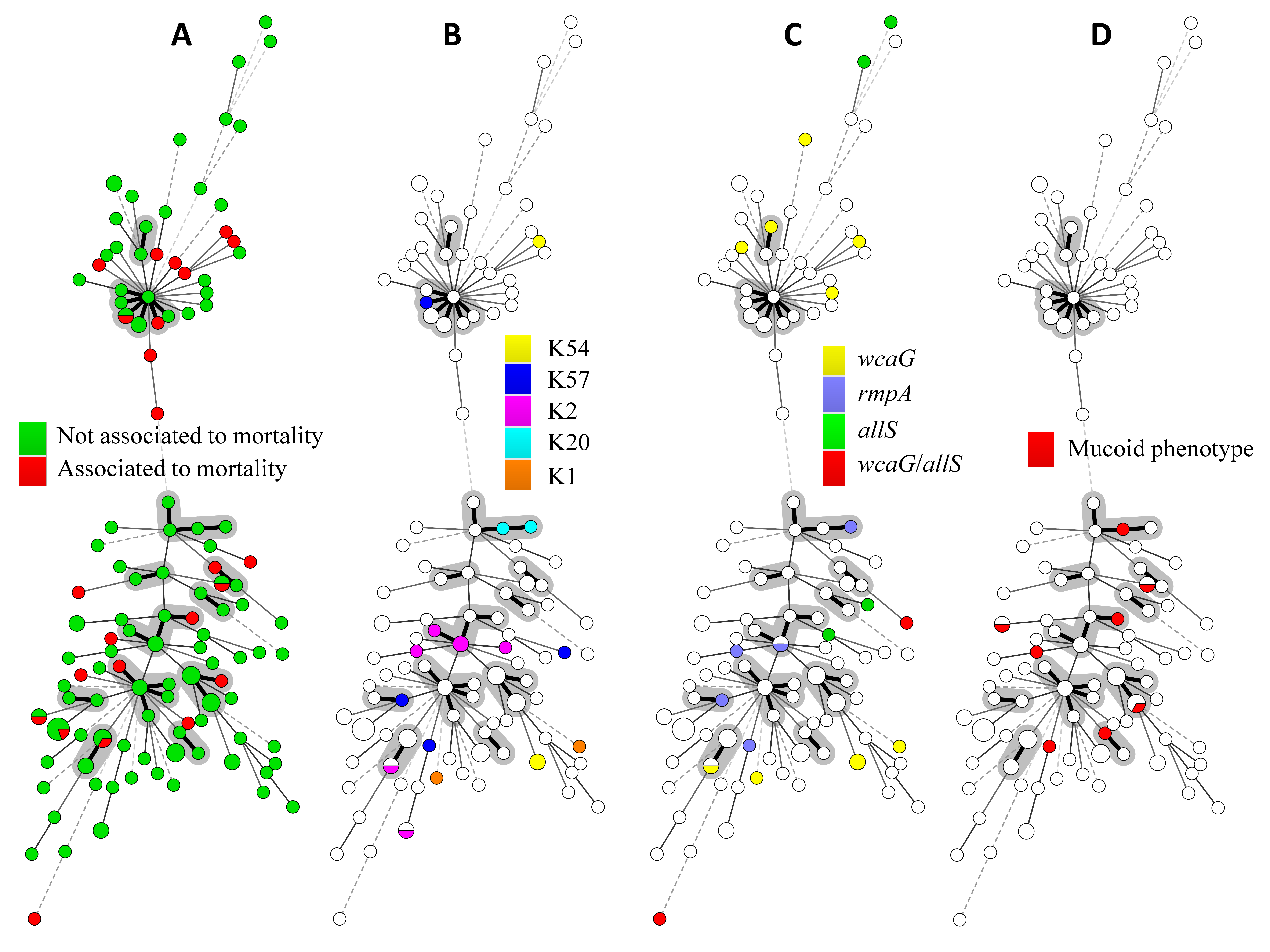

Supplement: Figure S2 — Minimal Spanning Trees (MSTs) analysis of Klebsiella pneumoniae strains based on MLST allelic profiles. Each circle corresponds to an ST. The area of each circle corresponds to the number of isolates. The relationships between strains are indicated by the connections between the isolates and the lengths of the branches linking them. Black lines connecting pairs of STs indicate that they differ in one allele (thick lines), two and three alleles (thin), or four to seven alleles (dashed). Four MST graphs were generated separately based on the following associations. A: MST vs mortality, B: MST vs serotypes, C: MST vs virulence genes and D: MST vs mucoid phenotype. (TIF) [file pone.0113539.s002.tif]
